# When Friends’ and Society’s Expectations Collide: A Longitudinal Study of Moral Decision-Making and Personality across College

**DOI:** 10.1371/journal.pone.0146716

**Published:** 2016-01-11

**Authors:** Kathryn L. Bollich, Patrick L. Hill, Peter D. Harms, Joshua J. Jackson

**Affiliations:** 1 Department of Psychology, Washington University in St. Louis, St. Louis, Missouri, United States of America; 2 Department of Psychology, Carleton University, Ottawa, Ontario, Canada; 3 Department of Management, University of Nebraska-Lincoln, Lincoln, Nebraska, United States of America; Georgia State University, UNITED STATES

## Abstract

Early adulthood is a developmentally important time period, with many novel life events needing to be traversed for the first time. Despite this important transition period, few studies examine the development of moral decision-making processes during this critical life stage. In the present study, college students completed moral decision-making measures during their freshman and senior years of college. Results indicate that, across four years, moral decision-making demonstrates considerable rank-order stability as well as change, such that people become more likely to help a friend relative to following societal rules. To help understand the mechanisms driving changes in moral decision-making processes, we examined their joint development with personality traits, a known correlate that changes during early adulthood in the direction of greater maturity. We found little evidence that personality and moral decision-making developmental processes are related. In sum, findings indicate that while moral decision-making processes are relatively stable across a four-year period, changes do occur which are likely independent of developmental processes driving personality trait change.

## Introduction

“Friends and loved ones are *special* to us; we do not, and should not, assess their interests from the cold detached standpoint of the impartial observer, for to do so would be a repudiation of love. No ethical system worth its salt will attempt to require such impartiality from us in these contexts, on pain of making human fulfillment unattainable.” [1]

Throughout their lives, people are confronted with decisions in which they have to choose whether it is more important to help a friend as opposed to following society’s rules. These grey areas have no right or wrong answer, with both poles offering an arguably ethical choice depending on one’s rationale and reasoning. For example, not turning in a friend caught cheating on an exam, or leniently grading a friend’s exam to ensure he passes, present a dilemma with arguably two ethical choices—being a loyal friend or an ethical employee. Because these choices have implications for society at large it is important to understand the developmental course of such decisions and what processes serve to influence and shape moral decision-making across time.

Unfortunately, little is known about the development of moral decision-making, particularly after childhood and adolescence. Post-adolescence marks a particularly critical period in which moral decision-making likely develops, as young adults begin to take on new roles outside their parents’ home, embark on new relationships, and engage in profound self and identity examination [2]. Using a four-year longitudinal study of young adults, the present research investigates the development of moral decision-making—specifically, what young adults choose when faced with conflicting obligations towards their friends and societal rules. Further, we examine potential reasons why development occurs by examining the joint development with personality traits. Personality traits are a known correlate of morality and their development is conceptually linked with moral development [3,4]. However, to date these two constructs have not been examined simultaneously over time.

### The Development of Moral Decision-Making

The development of moral decision-making has been described extensively through the work of Kohlberg and others [5–7] and tested in numerous studies [8,9]. However, few empirical studies extend past adolescence and use a longitudinal design. Those studies that have longitudinally examined post-adolescent samples suggest that people progress toward more sophisticated moral reasoning during college [10–13]. Some of this development may be unique to the college experience due to practices in collegiate settings that encourage more advanced moral development [14,15]. For instance, the deep learning approaches of some college classes that involve taking on divergent perspectives and integrating information from varied sources encourages growth in moral reasoning [16,17]. Overall, these studies indicate that young adulthood is particularly important with respect to moral development.

Unfortunately, these studies that have examined moral decision-making post-adolescence tend to lack in important ways. For example, previous examinations of college student moral development are often underpowered and occur over short periods of time, meaning researchers may be missing important aspects of development. Furthermore, these studies have not used sophisticated longitudinal models that would allow for assessing individual differences in development or shared development with other constructs. The current study sought to examine the development of moral decision-making by looking at three different ways to conceptualize change: rank-order stability, mean-level change, and individual differences in change [18]. To our knowledge this is the first study of moral development that has looked at these three types of change concurrently.

### Relationship Between Moral Decision-Making and Personality Traits

It is not fully known why moral decision-making may develop past adolescence. One potential means to help understand the mechanisms driving stability and change is to look at the development of characteristics associated with moral decision-making. The dispositional traits of agreeableness and conscientiousness regularly emerge as important characteristics of the moral individual across a number of studies [19–21]. Individuals who are agreeable are motivated to maintain positive peer relations and are sympathetic and warm [22–24], whereas individuals who are conscientious are organized, dependable, and rule-abiding, and show great self-control [25–27]. Across multiple cultures, moral persons or individuals of good character are attributed terms associated with agreeableness and conscientiousness, like “compassionate” or “responsible” [20,28–30], and individuals recognized for their moral dedication are perceived by themselves and others as having high levels of agreeableness and conscientiousness [19,21,31,32].

Despite a consistent link between personality and moral personhood, research is less clear on how personality traits may relate to moral decision-making processes. The majority of past research has focused on whether personality traits predict a broad, higher-level moral reasoning stage [33]. Typically, studies of this kind find that self-reported agreeableness and conscientiousness have little or no relationship to moral reasoning skill [34,35], which appears discrepant with the work on moral exemplars and individuals’ perceptions of moral individuals. However, this discrepancy may result from the moral reasoning measures employed in this work, like the Defining Issues Test [33]. The scenarios assessed in this measure may be too detached from what individuals face in their daily lives. This is in contrast to examining the more everyday moral behaviors that individuals may realistically encounter. The focus on higher-level thinking may obscure whether personality traits influence more relatable moral judgments that manifest in everyday behaviors [36].

Studies that have examined more common moral decisions and behaviors are more likely than DIT studies to implicate agreeableness and conscientiousness. For example, individuals who report lower levels of agreeableness and conscientiousness report engaging in more unethical Internet behaviors including fraudulence, misuse, and plagiarism [37]. Similar findings have been found within academic settings, with respect to agreeableness and conscientiousness predicting lower levels of academic cheating [38]. In both cases, though, the actor is the primary individual to benefit from potentially deviant behavior, not a close other. As such, it remains unclear whether agreeableness and conscientiousness relate to the decision to help a friend, when doing so is at odds with societal rules.

Given the nature of the two traits, it may be the case that agreeableness and conscientiousness actually have conflicting influences on such decisions. Specifically, individuals who are higher in conscientiousness may be more likely to adhere to societal rules than help a friend because of their rule-abiding nature and motivation to adhere to moral obligations [26,27,39]. In contrast, individuals who are higher in agreeableness may be more likely to help a friend than follow societal codes because of their sympathetic nature and motivation to maintain positive peer relations [22,23].

### The Development of Agreeableness and Conscientiousness

How individuals’ levels of agreeableness and conscientiousness develop over time may provide insight into how people respond to moral dilemmas. It is well-established that young adults, on average, tend to increase in agreeableness and conscientiousness [25,40–43]. These trends have been described as reflecting increases in maturity [42,43]. Longitudinal work also indicates that there are individual differences in these patterns [42]. That is, some people increase, others decrease, and still others stay consistent over time in their personality trait levels, including their levels of agreeableness and conscientiousness. Changes in personality traits are thought to coincide with changes in social roles [44,45] and an assortment of other social factors such as military experience [46].

No work to our knowledge has examined the role that personality trait development might play in the moral decision-making process and its development (or vice versa). Given that agreeableness, conscientiousness, and moral decision-making appear to change during the young adult years [11,47], one would expect these developments to coincide. Based on previous associations, as individuals increase in agreeableness, they likely will also increase their tendency to help their friend. Furthermore, as individuals increase in conscientiousness, they also should increase in the tendency to follow societal expectations.

### Current Study

The present study examined the relationship between moral decision-making and the personality traits of agreeableness and conscientiousness. We examined two components of the moral decision-making process. First, we were interested in one’s sense of *obligation* to a friend. That is, does a friend have the right to expect favors, when those favors are at odds with societal rules or expectations? Second, we examined one’s *decision* to help a friend. When faced with the dilemma between helping a friend or following societal rules, what do individuals choose? While many moral dilemmas pit “getting along” motives (e.g., helping others, sometimes at the cost of personal gain) and “getting ahead” motives (e.g., personal gain, sometimes at the cost of hurting others) against each other—which research proposes are two of humans’ most driving motives [48–50]—our measure presents a conflict between two getting along motives. This presents an understudied perspective on how people navigate moral obligations and decisions.

Our first aim was to assess whether these components of the moral decision-making process are related to morally relevant personality traits. Our second aim was to examine the longitudinal development of these components alongside morally relevant personality traits. To test these questions, we used a four-year longitudinal study of college student students who, during their freshman and senior years, completed measures of moral decision-making and personality.

## Method

### Sample and participants

The present study uses archival data from the Harvard Student Study [51] that were collected at Harvard University in the 1960s before institutional review boards and formal consent. Data were anonymized and de-identified prior to use. The sample consists of undergraduate students (freshman year: N = 667, *M*_*age*_ = 18.10 years, *SD*_*age*_ = .56) who were predominately White. At the time of data collection, Harvard only enrolled men. For the present study, 250 to 519 students completed relevant measures during their freshman year (Wave 1) and 197 to 376 students did so in their senior year (Wave 2). Sample size varied by analysis.

### Measures

#### Moral decision-making

Participants completed a variety of questionnaires and interviews over four consecutive years in college (for more information on measures collected, see work by Harms and colleagues) [52]. Of particular interest to the current study, participants completed a moral decision-making questionnaire [53] during their freshman and senior years. The questionnaire consisted of 16 hypothetical scenarios that pitted responsibility toward society (i.e., following socially prescribed rules) against allegiance to one’s peers. Example scenarios included asking participants whether: as a member of a company’s board of directors, they would share private financial information with a friend; as a drama critic, they would write a positive review for a friend whose play is not actually good; as an exam proctor, they would not turn in their friend whom they catch cheating; and as a test grader, they would leniently grade a friend’s exam to ensure that he passes.

For each scenario, participants (a) reported to what extent they believed they were expected or obligated to help their friend (*moral obligation*) on a 3-point scale (where ‘1’ reflected no obligation to friend, ‘2’ reflected some obligation to friend, ‘3’ reflected definite obligation to friend) and (b) reported whether they would behave in accordance with socially prescribed rules or behave in a manner that would benefit their friend (*moral decision*) on a dichotomous scale (where ‘1’ reflected not helping friend and ‘2’ reflected helping friend). As such, higher scores indicated greater feelings of obligation to a friend, and a greater number of decisions to help a friend rather than following societal expectations. However, we do not suggest that lower (or higher) scores indicate a more advanced level of decision-making ability, or that one choice is the decidedly 'moral' one in any of the scenarios. We instead employ the measure as an indicator of whether individuals focus more on helping their friends or on adherence to societal codes as their basis for making these decisions. Reliabilities for these two 16-item measures were satisfactory at both time points (moral obligation: freshman year α = .86, senior year α = .86; moral decision: freshman year α = .70, senior year α = .72). Although these two moral decision-making scales were related (freshman year *r* = .37; senior year *r* = .30), their correlation was modest enough to suggest they are distinct components of moral decision-making, and should be examined separately.

#### Personality traits

In addition to a measure of moral decision-making, participants rated themselves on a variety of adjectives. Latent agreeableness and conscientiousness factors were created for each participant at the two time points, factors that were based largely on previous factor analytic work by Harms and colleagues [52] who examined descriptive properties of the Harvard Student Study [51]. Following Harms’ and colleagues’ work with the present data, agreeableness was measured using four items from the Brownfain Self-Rating Inventory (i.e., trustfulness, understanding of others, sincerity, and generosity) [54] completed on an 8-point scale (1 = *low*, 8 = *high*), and two combined scales from the Myers-Briggs Type Indicator (i.e., the Feeling and the Thinking [reverse-scored] scales) [55,56] completed as a multiple choice test. The latent conscientiousness factor was created using four items from the Brownfain Self-Rating Inventory (i.e., initiative, dependability, neatness, and consistency) completed on an 8-point scale (1 = *low*, 8 = *high*), and two combined scales from the Myers-Briggs Type Indicator (i.e., the Judging and the Perceiving [reverse-scored] scales) completed as a multiple choice test. Although now outdated measures, Harms’ and colleagues’ work [52] as well as others’ [24,57,58] has determined that items from the Brownfain Self-Rating Inventory and the Myers-Briggs Type Indicator do an adequate job at capturing more modern measures of agreeableness and conscientiousness. We were not able to include items from the Omnibus Personality Inventory [59] as part of conscientiousness like Harms and colleagues did because it was not collected at Wave 1.

### Analyses

**Fig 1:**
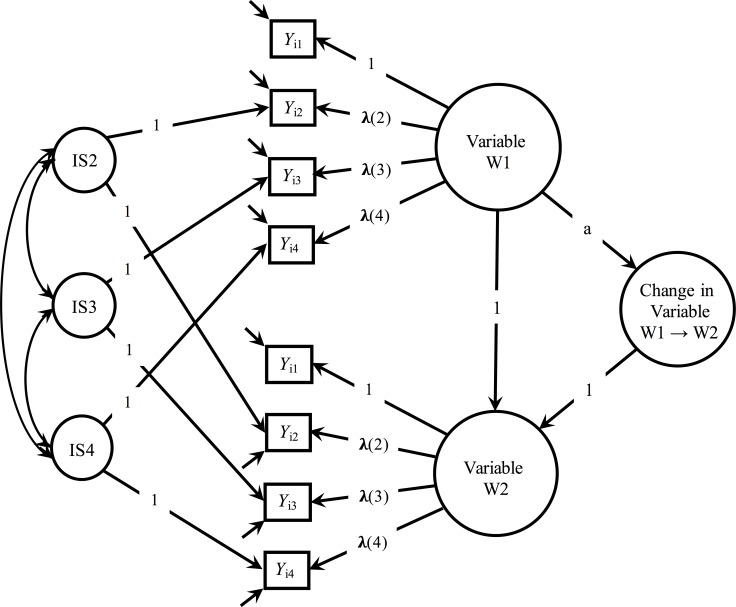
Example Measurement Model for Moral Decision-Making and Personality Measures. Moral decision-making models included four parcels as indicators (as depicted here). Personality models differed in that they included five indicators, which were not parcels. Loadings (λ) and intercepts (not displayed) were constrained to be equal over time for each indicator. Longitudinal method effects were accounted for by indicator-specific factors (IS). The path labeled “a” was freely estimated.

All analyses were run using R statistical software. Using structural equation modeling, we first tested univariate latent change models for each of our moral decision-making (moral obligation and moral decision) and personality constructs (agreeableness and conscientious) using the “lavaan” package in R [60]. See Fig 1. Model construction was based on McArdle’s work [61] and has been successfully used in previous studies of personality trait development [62]. For the moral obligation and moral decision constructs, we fit models using four parcels containing four items each; agreeableness and conscientiousness items were not formed into parcels. For each model, item indicators at each wave loaded onto latent factors that represent Wave 1 and Wave 2. These latent factors were then used to construct latent intercept and change parameters. The intercept factors were scaled to represent initial levels of the construct at freshman year while the change factor represented the changes in each construct between freshman and senior years. We fixed all item factor loadings and item residual variances to be equivalent across time points, and same item residuals were allowed to correlate across time.

Next, we fit four bivariate latent change models to assess the relationship between initial values and changes between each moral decision-making component and each personality trait [62]. Intercept and change parameters for personality traits and moral decision-making were allowed to correlate. In these bivariate models, three particular parameters of interest were estimated: intercepts of each construct correlated with one another; change factors regressed onto the intercept of the other factor; and both change factors correlated with one another. To assess fit, we examined the chi-square fit statistic (χ^2^), the comparative fit index (CFI), and the root mean square error of approximation (RMSEA). Data and full information on our analyses are available at https://osf.io/vxt6k/.

## Results

### Longitudinal Development of Moral Decision-Making

As shown in Table 1, moral obligations and decisions demonstrated high rank-order stability over the 4-year period (latent *r*s = .52 and .53, respectively). These analyses suggest that moral decision-making is a relatively stable construct across four years in young adulthood, though not at levels that would preclude changes in moral decision-making. In terms of cross-construct correlations, moral obligations during freshman year were modestly related to moral decisions during senior year (latent *r* = .14), and freshman moral decisions were modestly related to moral obligations during senior year (latent *r* = .21). These results provide support for these two components of moral decision-making to be considered separately in our analyses.

**Table 1:** Intercorrelations Among Latent Measures of Moral Decision-Making and Personality.

Construct	1	2	3	4	5	6	7
1. Moral Obligation *(Freshman Year)*	*-*	-	-	-	-	-	-
2. Moral Decision *(Freshman Year)*	.41*	*-*	-	-	-	-	-
3. Agreeableness *(Freshman Year)*	.07	.04	*-*	-	-	-	-
4. Conscientiousness *(Freshman Year)*	-.09	-.26*	.35*	*-*	*-*	*-*	*-*
5. Moral Obligation *(Senior Year)*	.52*	.21*	.01	-.04	-	-	-
6. Moral Decision *(Senior Year)*	.14*	.53*	-.14	-.31*	.39*	-	-
7. Agreeableness *(Senior Year)*	-.03	.02	.70*	.20	-.12	-.08	-
8. Conscientiousness *(Senior Year)*	-.09	-.04	.33*	.69*	-.14	-.22*	.52*

* = *p* < .05.

Next, we examined mean-level changes in obligations and decisions. Both of the longitudinal models fit the data well (Table 2; standardized item loadings ranged from .602 to .854 at Wave 1 and .651 to .844 at Wave 2 for obligations, and .410 to .758 at Wave 1 and .429 to .798 at Wave 2 for decisions). Our sample experienced mean-level changes in moral obligations, such that over the 4-year period, participants on average felt more obligated to help a friend rather than follow the rules of society (est. = .552, *SE* = .087, *t* = 6.317, *p* < .001). Similarly, our sample experienced increases in the tendency to decide to help a friend instead of following societal expectations (est. = .402, *SE* = .101, *t* = 3.974, *p* < .001).

**Table 2:** Means and Variances for Intercepts and Slopes of Moral Decision-Making and Personality Measures.

	Intercept		Slope		Model Fit Statistics			
Construct	Est. (SE) [95% CIs]	Variance (SE) [95% CIs]	Est. (SE) [95% CIs]	Variance (SE) [95% CIs]	χ^2^	df	RMSEA	CFI
Moral Obligations	1.550* (.016) [1.518, 1.582]	.096* (.009) [.078, .114]	.552* (.087) [.380, .723]	.047* (.007) [.032, .061]	52.362	16	.066	.977
Moral Decisions	1.482* (.011) [1.462, 1.503]	.027* (.004) [.019, .035]	.402* (.101) [.204, .601]	.009* (.002) [.004, .013]	48.001	16	.062	.961
Agreeableness	5.862* (.085) [5.695, 6.029]	.827* (.184) [.466, 1.187]	.278 (.448) [-.600, 1.156]	.158 (.130) [-.096, .413]	28.067	28	.002	1.000
Conscientiousness	5.477* (.099) [5.283, 5.672]	1.762* (.288) [1.197, 2.326]	1.174* (.433) [.326, 2.022]	.354† (.182) [-.003, .710]	41.461	28	.031	.972

* = *p* < .05

† = *p* < .10

Est. = unstandardized estimate, SE = standard error, 95% CIs = 95% confidence intervals.

Not everyone followed this pattern of mean-level changes though. As indicated by the variance estimates for the slope estimates, there were individual differences in change patterns for both moral obligations (est. = .047, *SE* = .007, *t* = 6.366, *p* < .001) and moral decisions (est. = .009, *SE* = .002, *t* = 3.80, *p* < .001). Thus, while the general trend was to increase one’s tendency to help out a friend in need, not everyone changed to the same degree.

### Longitudinal Development of Personality Traits

Changes in agreeableness and conscientiousness across college were examined next. Across four years, both agreeableness and conscientiousness evidenced high rank-order stability (latent *r*s = .70 and .69, respectively; Table 1). Longitudinal models indicated a good fit to the data (Table 2; standardized item loadings ranged from .328 to .639 at Wave 1 and .101 to .658 at Wave 2 for agreeableness, and .306 to .632 at Wave 1 and .257 to .662 at Wave 2 for conscientiousness). In terms of mean-level change, participants did not change in their levels of agreeableness (est. = .278, *SE* = .448, *t* = .620, *p* = .535), but did increase in their levels of conscientiousness (est. = 1.174, *SE* = .433, *t* = 2.713, *p* = .007). There was some evidence for individual differences in change as evidenced by the variance components for conscientiousness (est. = .354, *SE* = .182, *t* = 1.943, *p* = .052) but there was less evidence for individual differences in agreeableness (est. = .158, *SE* = .130, *t* = 1.221, *p* = .222).

### Joint Longitudinal Development of Moral Decision-Making and Personality Traits

First, we examined associations between the moral decision-making components and personality traits. All models fit the data well (χ^2^s ≤ 151.223, RMSEAs ≤ .031, CFIs ≥ .965). In examining the cross-sectional relationships among moral decision-making and personality (Table 1), we found that moral obligation was not strongly associated with agreeableness or conscientiousness (freshman year: latent *r*s = .07 and -.09, senior year: latent *r*s = -.12 and -.14, for agreeableness and conscientiousness, respectively). Similar findings were found for moral decisions (freshman year: latent *r*s = .04 and -.26; senior year: latent *r*s = -.08 and -.22, for agreeableness and conscientiousness, respectively).

Next we investigated the joint development of these constructs using bivariate latent change models (Table 3). First, we examined whether initial levels of personality were associated with the tendency to increase or decrease in the likelihood of helping a friend. No meaningful cross-lagged results emerged. We next tested whether initial levels of moral decision-making predicted changes in personality. We found that freshman levels of moral decisions were marginally associated with increases in conscientiousness (est. = .741, *SE* = .446, *t* = 1.662, *p* = .097). No other cross-lags evidenced meaningful relationships.

**Table 3:** Bivariate Latent Change Models.

	Personality Intercept & Moral Intercept		Personality Intercept & Moral Slope		Moral Intercept & Personality Slope		Personality Slope & Moral Slope	
Bivariate Model	Est. (SE)	[95% CIs]	Est. (SE)	[95% CIs]	Est. (SE)	[95% CIs]	Est. (SE)	[95% CIs]
Moral Obligation & Agreeableness	.007 (.031)	[-.053, .067]	.047 (.031)	[-.014, .108]	-.143 (.168)	[-.472, .186]	-.007 (.006)	[-.019, .004]
Moral Obligation & Conscientiousness	.036 (.035)	[-.033, .105]	.003 (.015)	[-.026, .032]	.001 (.221)	[-.433, .435]	-.015† (.008)	[-.032, .001]
Moral Decision & Agreeableness	-.032 (.021)	[-.074, .009]	.010 (.018)	[-.025, .045]	-.065 (.381)	[-.813, .682]	-.001 (.003)	[-.006, .005]
Moral Decision & Conscientiousness	.008 (.023)	[-.037, .053]	-.009 (.008)	[-.025, .007]	.741† (.446)	[-.133, 1.614]	-.006 (.003)	[-.012, .001]

† = *p* < .10

Est. = unstandardized estimate, SE = standard error, 95% CIs = 95% confidence intervals. The first column of results displays the relationships between personality traits and the moral decision-making components’ intercepts. The second column of results displays the relationships between personality trait intercepts with changes in the moral decision-making components. The third column of results displays the relationships between the moral decision-making components’ intercepts with changes in personality traits. The fourth column of results displays the relationships between changes in personality traits and moral decision-making components from freshman year to senior year.

Lastly, we examined whether changes in obligations and decisions were associated with changes in agreeableness and conscientiousness (Table 3). Changes in moral obligation were slightly related to changes in conscientiousness (est. = -.015, *SE* = .008, *t* = -1.873, *p* = .061), such that individuals who increased in their obligations to societal code also increased in their conscientiousness. No other meaningful relationships were found.

## Discussion

The present study examined moral decision-making in the context of dilemmas in which individuals had to decide between breaking the rules of society to help out a friend or following societal rules at the consequence of negatively affecting that friend. Specifically, we were interested in how responses to these dilemmas develop across young adulthood, and whether moral decision-making was associated with personality traits and personality trait development. Our findings advance and support previous research suggesting that moral decision-making continues to develop past childhood and adolescence and into the college and young adult years [11] while still showing considerable stability. In addition, these findings extend work examining the overlap between personality traits and moral decision-making [34,35] by examining their co-development. Our results suggest little joint development, indicating that moral decision-making and personality trait development processes are largely separable from one another [34]. As such, it is important to consider both types of variables separately when considering the complexities of moral personhood, and to extend work to other individual differences that may play a closer role in the development of moral decision-making.

### Moral Decision-Making as a Relatively Stable Trait

Our study provides support for moral decision-making as a stable individual difference across a four-year period. Much work in morality focuses on the influence of the situation and neglects the predictive power of stable, individual characteristics for influencing moral judgments and behaviors [63–65]. The rank-order stability correlations for moral obligation and moral decisions found here are indeed quite similar to the stability estimates for personality traits at this age [66]. These findings fit well with recent work showing the moderate stability of morality in everyday life and across different situations [36,67], providing additional evidence for the existence of a relatively stable moral character. While situational conceptualizations of moral character are indeed important, these studies and the present findings highlight the need to incorporate relatively stable individual differences into theories of moral behavior [3].

Past longitudinal research on moral development has largely focused on children and adolescents, whereas our findings support previous research in suggesting that the young adult years—when people begin redefining themselves and creating a separate identity from their familial and hometown ties—also prove a crucial period for development [10,11]. Specifically, young adults in our study, on average, increased in their feelings of obligation to close others and were more likely to decide to help them rather than follow socially prescribed rules. Considered in the context of competing “getting along” goals [50], it is interesting that getting along with peers became stronger than the desire to get along with societal expectations throughout the college years, and raises the question of how competing “getting ahead” goals might change during these years. These findings also raise the question of what mechanisms drive changes in moral decision-making, as currently few studies exist that investigate the natural development of moral decision-making during young adulthood or that attempt to identify experiences that influence moral development. One mechanism of particular importance may be changes in what people value with respect to their social networks. Specifically, people tend to create a smaller social network as they age, and their social network increases in importance [68,69]. Assessing whether changes in moral decision-making are specific to the college age or are only the beginning of this normative trend to increasingly help out friends rather than follow societal codes will be helpful for understanding the mechanisms underlying these changes.

### Moral Decision-Making Development and Personality Traits

Our results also suggest that changes in moral decision-making practices during adulthood are likely largely independent from the processes that drive personality development. Changes in conscientiousness and agreeableness during the early adulthood years are characterized as evidence for increases in maturity, as agreeableness is associated with being kind and warm, and conscientiousness reflects the ability to control one’s impulses [41]. As such, it is relatively surprising that these processes do not show greater overlap. This may be due in part to our measures used. For example, researchers might benefit from focusing on the various facets of personality. For instance, these moral dilemmas may actually cause two aspects of agreeableness—compliance and empathy—to be in conflict, thus obscuring the unique and contrasting influences both may play in moral decision-making [22]. It is also possible that agreeableness may play a bigger role in the development of non-deliberative moral action, for example, caring for others and other prosocial behaviors [22].

Other individual differences and developmental processes may prove more fruitful in understanding the processes behind changes in moral decision-making. For example, major life experiences that tend to increase one’s focus on the well-being of close others, such as marriage, child-rearing, and caring for older relatives [70], offer another important avenue for explaining stability and change in moral decision processes. Life goals may be another important individual difference to consider. Changes in life goals—for example, focusing on family over work—influence what life roles people find important and people then subtly changing their daily behaviors and values in order to achieve those goals [71]. If one’s goals shift toward a more communal orientation (e.g., friends and family members over personal advancements), someone is likely to change one’s moral decisions given the greater value placed on friends and family members. Future work that examines the goals and motives associated with different events and stages of development may help to explain developmental process leading to changes in moral decision-making.

### Limitations, Future Directions, and Conclusions

It is necessary to note aspects of the current study that should serve as catalysts for future research. First, our longitudinal sample was predominately White and affluent, and attended an elite all-male school in the early 1960s. Future work thus should examine the generalizability of our findings to women, more traditional collegiate contexts, and non-collegiate samples. Second, our measure of moral dilemmas has received little empirical investigation [72–74] and its ability to predict moral behavior is unclear. Considering the evaluativeness of morality, this self-report of moral decision-making may be especially prone to biased self-report [75]. Ideally, future work will include more objective moral measures, rather than only self-reported decisions and behaviors [36]. In addition, although our personality measure has been validated in previous work [52], using more current personality measures would be useful, especially in order to examine how facets of agreeableness (e.g., sympathy vs. modesty) and conscientiousness (e.g., dutifulness vs. self-discipline) might relate differently to moral decision-making.

Taken together, the present study provides clear evidence that moral decision-making continues to develop past childhood and adolescence, and confirms and extends previous work suggesting that personality has little cross-sectional or longitudinal relationship to moral decision-making. Research would benefit from examining the longer-term implications of early adult experiences on later moral decisions and behaviors, as well as how moral decision-making may continue to change in later adult years. Furthermore, it is sometimes questionable what direction of changes in moral decisions and obligations should be considered a positive or mature development. These grey areas are a particularly intriguing aspect of morality research and are important to consider, especially when the goal of moral development research is often focused on understanding the “improvement” of moral character. Always siding with societal standards or always choosing friends’ needs may actually be the least mature option, as neither may be adaptive.

In closing, if it is true, as John Cottingham states in our opening quote, that close others cannot be evaluated from a “cold detached standpoint,” it is important to gain a better understanding of which individuals are especially prone to choosing friends over society and what factors contribute to their decision, especially when there may be large repercussions resulting from that decision.
